# Ubiquitin-Specific Protease 2 (USP2) as a Modulator of Energy Metabolism: A Review of Studies Using Animal and Cellular Models

**DOI:** 10.3390/biomedicines14040783

**Published:** 2026-03-30

**Authors:** Hiroshi Kitamura, Jun Okabe, Himeka Hayashi, Tomohito Iwasaki

**Affiliations:** 1 Department of Laboratory Animal Medicine, Graduate School of Medicine, Tohoku University, Sendai 980-8575, Japan; himeka.hayashi.d6@tohoku.ac.jp; 2 Epigenetics in Human Health and Disease Laboratory, Baker Heart and Diabetes Institute, Melbourne, VIC 3004, Australia; jun.okabe@baker.edu.au; 3 Department of Food Science and Human Wellness, Rakuno Gakuen University, Ebetsu 069-8501, Japan; iwasaki@rakuno.ac.jp

**Keywords:** USP2, ubiquitination, proteasome, obesity, insulin tolerance, diabetes mellitus, atherosclerosis, MASLD, male infertility, oncogenesis

## Abstract

Ubiquitin-specific protease 2 (USP2) is a deubiquitinase that controls various cellular events, including cell cycle progression and tumorigenesis. Along with cell culture models, mouse models induced using chemical blockers and gene engineering have substantially contributed to our knowledge of the crucial roles of USP2 in energy metabolism and metabolic disorders. This review summarizes the evidence of the role of USP2 in regulating energy metabolism in mice and cells under physiological and pathological conditions. In hepatocytes, a short isoform of USP2, USP2b, aggravates type 2 diabetes and metabolic dysfunction-associated steatotic liver disease. Meanwhile, a long isoform of USP2 in adipose tissue macrophages, USP2a, attenuates the onset of diabetes. USP2a mitigates insulin resistance and subsequent muscle atrophy. In ventromedial hypothalamic neurons, USP2b inhibits an increase in blood glucose by repressing hepatic glycogenolysis. In addition to regulating diabetes, USP2 isoforms potentially regulate the progression of atherosclerosis by modulating macrophages and hepatocytes. In brown adipose tissue, USP2a regulates thermogenesis, thus influencing systemic energy control. Meanwhile, in testicular macrophages, USP2 protects the mitochondrial respiration of sperm and consequently contributes to maintaining the quality of frozen sperm for use in the treatment of male infertility. As USP2 is distributed to multiple cellular components, it mediates the polyubiquitination of various molecules. For instance, USP2 modulates the stability of various transcription regulators, including C/EBP-α, PPARγ, EBF2, and PGC1α. The accumulating evidence indicates that USP2 functions as a modulatory molecule for energy metabolism across organs.

## 1. Introduction

In energy metabolism, the first priority is the generation of ATP, which is the common energy currency of cells. In eukaryotes, ATP is mostly produced from glucose via glycolysis in the cytoplasm and oxidative phosphorylation (OXPHOS) in mitochondria [1,2]. Since energy depletion is fatal for cells and organisms, multiple mechanisms at various levels operate in conjunction to maintain sufficient ATP supply. During periods of glucose deficiency, cells utilize amino acids, glycerol, and lactate through gluconeogenesis [3,4]. In humans, following starvation for 14 h, gluconeogenesis provides nearly 50% of circulating glucose. Meanwhile, following starvation for 42 h, gluconeogenesis provides >90% of circulating glucose [5]. Fatty acids are another crucial source of energy during periods of fasting [2]. Together, the carbohydrate and lipid metabolisms are estimated to provide >90% of the body’s energy requirements [2].

The problem of overnutrition is mitigated by multiple mechanisms. Insulin secreted by pancreatic β-cells lowers blood glucose by enforcing glucose uptake by energy metabolism-competent organs and tissue types such as the liver, skeletal muscle, and adipose tissue [6]. Leptin from lipid-laden adipocytes attenuates the appetite and promotes energy consumption by activating the sympathetic nervous system [7,8]. However, regular consumption of a Western diet involving excessive intake of saturated fat, cholesterol, and sugar and insufficient intake of fiber and minerals causes energy homeostasis to break down [9,10]. The oversupply of saturated fat and carbohydrates eventually causes the deposition of energy as triglycerides in adipose tissue, resulting in obesity [11]. Enlarged adipose tissue secretes several detrimental adipokines, including resistin, tumor necrosis factor (TNF)-α, and interleukin (IL)-6, thus promoting local inflammation and systemic insulin resistance [11,12]. Obesity is a major risk factor for hypertension, as it enhances the activity of both the sympathetic nervous system and the renin–angiotensin–aldosterone axis [13]. High levels of circulating free fatty acids (FFAs) adversely affects both insulin resistance [14] and insulin signaling in the skeletal muscle [15]. Disrupted energy balance is a primary driver of type 2 diabetes (T2DM). While T2DM is a fundamental condition underlying diabetic complications such as kidney disease, retinopathy, and sarcopenia, it is also a risk factor for other metabolic diseases such as atherosclerosis and metabolic dysfunction-associated steatotic liver disease (MASLD), the latter of which has largely replaced the former term non-alcoholic fatty liver disease (NAFLD) [16,17,18,19]. In 2021, more than 500 million people globally (~5.9% of the global population) suffered from T2DM [20]. During the years 2016–2019, 38% of adults were predicted to have MASLD, while its prevalence is expected to increase to 55.4% by 2040 [19]. There is therefore a critical need for therapies that can normalize energy metabolism. Many drugs targeting energy metabolism-related molecules have been developed to treat metabolic diseases, such as peroxisome proliferator-activated receptor (PPAR)-γ, sterol regulatory element-binding proteins (SREBPs), and mechanistic target of rapamycin complex 1 (mTORC1) [21,22,23]. Nonetheless, innovative approaches targeting novel targets are required to establish more efficient treatment strategies. As a preliminary step, the molecules that are critical in regulating energy metabolism in various tissues have been examined.

Ubiquitination—the process whereby ubiquitin, a protein comprising 76 amino acids, is covalently bound to a target—is widely used to chemically modify proteins [24]. Ubiquitin has seven lysine residues (K6, -11, -27, -29, -33, -48, and -63), which conjugate to the methionine 1 residue to create ubiquitin chains [24]. K48-linked polyubiquitination, the most common type of polyubiquitination, serves as a trigger for protein degradation by the 26S proteasome [25]. In contrast, K63-linked polyubiquitination modulates protein–protein interactions, altering cellular functions such as signal transduction and intracellular trafficking [26]. Ubiquitination is reversibly regulated by ubiquitin ligases and deubiquitinating enzymes (DUBs), of which there are two types: cysteine proteases and metalloproteases [27]. Cysteine proteases called ubiquitin-specific proteases (USPs) form the largest family of DUBs. Based on comprehensive analyses, the human genome includes 58 USPs ranging in size from 50 to 300 kDa [27,28]. The structural conservation of USPs has been observed within their catalytic domain, known as the USP domain [28]. The canonical USP domain comprises unique palm–thumb–finger subdomains [29]. The finger subdomain captures ubiquitin, while there is a catalytically active site (the C-H-D/N catalytic triad) at the interface between the palm and thumb subdomains [28].

In a recent proteomics-based analysis, diabetic rats exhibited changes in the ubiquitination of hepatic proteins involved in glucose and lipid metabolism, providing evidence that protein ubiquitination affects overall energy metabolism [30]. Given that protein ubiquitination determines the abundance of target proteins, the ubiquitination of metabolic enzymes or their modulators will profoundly affect energy metabolism. Consistent with this, several USPs have been found to be metabolically important [31]. In adipocytes, USP1 promotes adipogenesis by stabilizing CCAAT/enhancer-binding protein (C/EBP) β, leading to the aggravation of obesity and insulin resistance [32]. USP38 is a detrimental factor of diabetic cardiomyopathy that accompanies abnormal lipid metabolism [33], while USP28 protects mitochondrial dysfunction in the diabetic heart [34]. In high-fat diet (HFD)-fed mice, hepatic USP20 maintained levels of HMG-CoA reductase, the rate-limiting enzyme of cholesterol synthesis, thus exacerbating hyperlipidemia, obesity, and insulin resistance [35]. Compared to the metabolic functions of other USPs, those of USP2 in various tissue types are relatively well understood. This review focuses on the roles of USP2 in both the cellular and systemic energy metabolisms. In particular, we highlight the value of animal and cellular models in elucidating USP2-modulated molecular events. To identify the references relevant to the regulation of energy metabolism by USP2, we surveyed all 320 articles referring to USP2 in PubMed (final search date: 20 February 2026). From these, we selected 17 articles that described the roles of USP2 in glucose and lipid metabolism at both organismal and cellular levels. We also identified ten studies that reported the involvement of USP2 in metabolic diseases such as T2DM, MASLD, and atherosclerosis. In addition, we collected 32 articles that examined the function of USP2 in the liver, skeletal muscle, adipose tissue, and hypothalamus. After removing duplicates, a total of 51 articles remained; from these, we further extracted 19 reports that directly demonstrated the involvement of USP2 in energy metabolism or metabolic disorders (Table 1 and Table 2).

In this review, we first provide an overview of the history, structure, and general functions of USP2. We then summarize the roles of USP2 in each tissue, mainly based on the 19 key studies identified. Furthermore, we present a comparative analysis of USP2-modulated events across tissues and discuss the limitations of current USP2 research.

## 2. Overview of USP2

**Figure 1:**
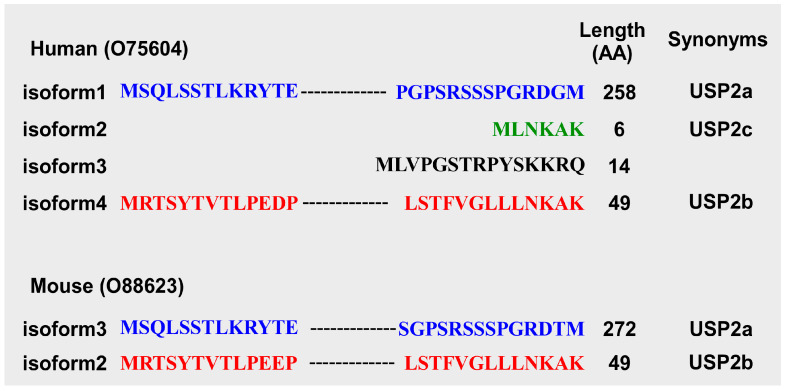
Different N-terminal extensions of USP2 alternative-splicing variants in humans and mice. Isoform numbers correspond to human (O75604) and mouse (O88623) USP2 entries in UniProt. Based on amino acid sequence comparisons, O75604-1 appears to be the orthologue of O88623-3 (blue), whereas O75604-4 appears to be the orthologue of O88623-2 (red). In humans, two additional isoforms are also annotated: O75604-2 (green) and O75604-3 (black). The length of each N-terminal extension recorded in UniProt is shown. Synonyms based on a previous paper [63] are also indicated. Notably, another USP2 variant known as O86623-3 has a similar N-terminal region to O86623-2, although it lacks three amino acids in the C-terminal of the extension. AA, amino acids.

Using cDNA cloning, USP2 was originally identified in the skeletal muscle of chickens in 1997 as ubiquitin-specific processing protease (UBP) 41 [55]. The first paper on mammalian USP2 reported two major splicing variants: the novel testes-specific UBP-t1 and UBP-t2. These variants differ in their N-terminal structures due to alternative splicing [56]. The major longer (~60 kDa) and shorter (~41–45 kDa) variants of USP2 were later referred to as USP2a and USP2b [57,58], UBP69 and UBP41 [59,60], or USP2-69 and USP2-45 [36,44,61], respectively. In the UniProt database, human USP2a (605 amino acids) and USP2b (353 amino acids) are deposited as O75604-1 (isoform 1) and O75604-2 (isoform 2), respectively (Figure 1). Two other variants also occur in humans: O75604-3 (isoform 3, 362 amino acids) and O75604-4 (isoform 4, 396 amino acids) [62]. In contrast, for USP2 in mice, only orthologues of human isoforms 1 and 4 are deposited in UniProt, as O88623-3 (619 amino acids) and O88623-2 (396 amino acids), respectively. Although O75604-2 is registered as “USP2b” in UniProt, it has previously been referred to as “USP2c” [63], creating confusion about whether human USP2b corresponds to O75604-2 or O75604-4. To avoid this inconsistency, we followed the nomenclature used in the previous report [63]: O75604-1 and O88623-3 are designated as USP2a, O75604-4 and 88623-2 as USP2b, and O75604-2 as USP2c in this review (Table 1). Human and mouse transcripts encoding USP2a, USP2b, and USP2c are described as *USP2a*/*Usp2a*, *USP2b*/*Usp2b*, and *USP2c*/*Usp2c*, respectively. In cases where the original articles did not specify which isoform was used, we inferred the isoforms based on the information provided in the Materials and Methods sections, such as primer sequences and expression constructs. When the isoform could not be clearly determined, we explicitly stated “isoform not specified”.

All USP2 variants share a conserved USP domain of 347 amino acids at their C-terminal [61,62]. In USP2 and the other USPs, the USP domain exhibits a palm–thumb–finger scaffold [64,65]. The catalytic triad of USP2 (C276, H557 and N574) and the zinc-binding motif (C425, C428, C477 and C479) have been predicted [65,66]. The N-terminal extensions of USP2 variants bind specific proteins, achieving intracellular localization and substrate accessibility [44,56]. ML364, a small-molecule inhibitor, is widely used to inhibit USP2 activity in vivo and in vitro [41,47,67,68,69,70]. The recombinant catalytic domain of USP2 has been used to explore the ubiquitination sites of 3338 proteins [71]. The USP domain of USP2 therefore has the potential to digest the polyubiquitin chain of a wide variety of proteins.

USP2 controls an exceptionally diverse range of biological processes [31,72]. It modulates periodic cellular processes such as those involved in cell cycle progression [73,74,75] and the circadian rhythm [76,77,78]. USP2 isoforms maintain the stability of brain and muscle Arnt-like protein 1 (BMAL1) and period1 (PER1), which regulate the circadian rhythm [77,79]. USP2 isoforms also maintain the stability of cyclin A1 and cyclin D1, thus affecting cell cycle progression [73,80,81]. USP2a has positive and negative impacts on inflammatory cytokine production, based on the findings of cellular and animal models. For instance, in macrophage-like HL60 cells, USP2a attenuated lipopolysaccharide-induced pro-inflammatory cytokine production by modulating the binding ratio of octamer transcription factor (Oct)-1 to Oct-2 at the cytokine promoters [57], whereas it augmented TNF-α-induced chemokine production in HeLa cells, presumably by altering nuclear factor-κB (NF-κB) signaling [82]. Under normal growth conditions, *Usp2* knockout (KO) mice normally grow up, except for those with male sterility [48], suggesting that USP2 only functions in specific situations. Consequently, many of the recent reports on the roles of USP2 have addressed diseases and conditions such as cancer, inflammation, and others involving stress [67,72,83,84].

## 3. Physiological and Pathological Roles of USP2 in Energy Metabolism in Several Cells and Tissues

### 3.1. Human Genome Data

Human genome data indicate the possible involvement of USP2 in the onset of metabolic diseases. For instance, Human Genetic Evidence (HuGE) scores from the Common Metabolic Diseases Knowledge Portal (http://hugeamp.org) predict that the *USP2* gene is “Very strongly” linked to “Weight” and “Body mass index (BMI)”. Bioinformatics of the a microarray data [GSE154337 in Gene Expression Ominibus (GEO)] reveal that *USP2* is one of the “top10 hub ubiquitination-related genes” in the protein–protein networks of patients suffering from gestational diabetes, and transcription factor–mRNA–miRNA network analysis indicates that the USP2 protein is a key ubiquitination regulator in the disease [85]. Moreover, RNA sequencing analysis of human liver data (GSE135251) shows that the expression level of *USP2* is significantly higher in MASLD patients than healthy controls [38]. These human genome data suggest the modulatory roles of USP2 in the onset of human metabolic diseases.

### 3.2. Hepatic USP2

The liver contributes to glucose homeostasis predominantly via glycogenesis, glycogenolysis, glycolysis, and gluconeogenesis [86]. Although the liver contains one-fourth of the glycogen content of skeletal muscle in humans [87], it is a major source of circulating glucose during fasting conditions. It produces glucose via glycogenolysis in the early phase of fasting (before 30 h) [86] and produces glucose from protein, lactate, and glycerol via gluconeogenesis once glycogen is depleted [3]. The liver controls blood glucose levels via the biosynthesis of cortisol, which is originally synthesized in the adrenal cortex and is converted to inactive cortisone by 11β-hydroxysteroid dehydrogenase 2 (11β-HSD2), primarily in the collecting ducts of the kidneys [88]. Conversely, 11β-HSD1 converts cortisone into cortisol in the liver, thereby promoting insulin resistance, fatty liver, and hypertension [89]. Accordingly, the inhibition of hepatic 11β-HSD1 has been proposed as an effective target for T2DM therapy [90].

**Figure 2:**
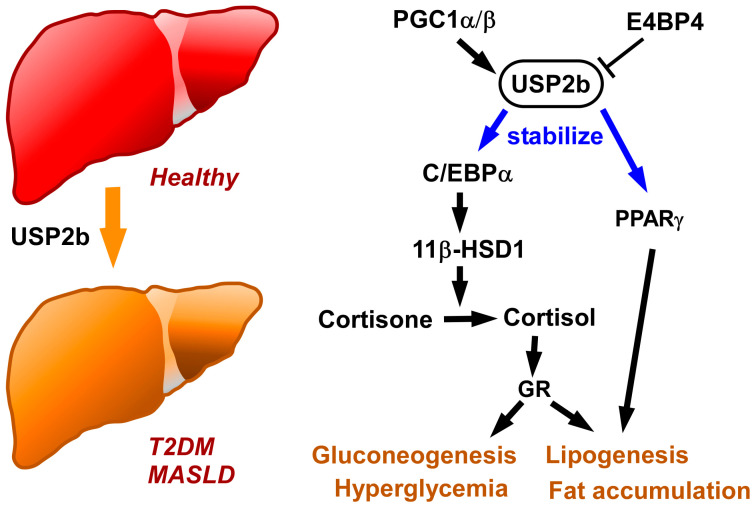
Putative roles of hepatic USP2b in the pathogenesis of T2DM and MASLD. (**Left**): Hypothetical scheme illustrating the roles of hepatic USP2b in the onset of T2DM and MASLD. (**Right**): Upstream and downstream molecules regulating or regulated by USP2b in the liver. PGC1α/β and E4BP4 transcriptionally up- and downregulate the *Usp2b* gene, respectively. USP2b stabilizes the transcription factors C/EBPα and PPARγ, presumably through deubiquitination. Increased C/EBPα induces 11β-HSD1, which converts inactive cortisone into active cortisol. Consequently, the cortisol-glucocorticoid receptor (GR) complex activates the transcription of genes involved in gluconeogenesis and lipogenesis. In parallel, increased PPARγ expression promotes the expression of the genes used for lipogenesis in hepatocytes. This figure was illustrated based on the findings reported in previous studies [36,37,38].

The physiological and pathological roles of USP2 in the liver are relatively well understood (Figure 2). Although USP2 isoforms mitigate hyperglycemia in other tissue types, hepatic USP2b adversely affects glucose metabolism. In mice, the abundance of hepatic *Usp2b* mRNA exhibits a diurnal pattern, reaching the peak at the dark phase and gradually returning to baseline at the light phase, while changes in *Usp2a* mRNA were marginal [36]. Peroxisome proliferator-activated receptor γ coactivator 1 (PGC1) α and β positively regulate *Usp2b* expression in cultured mouse hepatocytes, while E4-binding protein 4 (E4BP4) negatively regulates *Usp2b* expression [36,91].

As mentioned above, hepatic USP2b elevates blood glucose levels. In mice, USP2b exacerbated insulin resistance and glucose intolerance in HFD-fed mice [36]. USP2b positively regulates the gene expression of several enzymes used in energy metabolism, including phosphoenolpyruvate carboxykinase (PEPCK), glucose 6-phosphatase (G6Pase), glucose-6-phosphate translocase, and fatty acid synthase (FASN) [36]. Accordingly, the expression level of *Usp2b* determines hepatic glycogen content [36]. Strikingly, the perturbation of hepatic *Usp2b* expression alters fasting blood glucose and insulin levels, as well as pyruvate tolerance, even in lean mice [36]. Therefore, hepatic USP2b has modulatory effects on systemic glucose metabolism, even under steady-state conditions.

Since USP2b maintains 11β-HSD1 protein level, USP2b might potentiate conversion from cortisone to cortisol in the liver [36]. Inhibition of 11β-HSD1 represses the hepatic expression of *Pepck1* and *G6pase* as well as blood glucose levels, all of which were restored by *Usp2b* overexpression [36]. Thus, the synthesis of cortisol by 11β-HSD1 therefore seems to be crucial for USP2b-evoked glucose overflow from the liver [36]. USP2 has been suggested to stabilize C/EBPα [36], a putative transcription activator of *Hsd1* genes in hepatocytes [36,92]. Based on this evidence, hepatic USP2b was speculated to induce cortisol-dependent hyperglycemia by stabilizing C/EBPα.

In addition to affecting glucose metabolism, the liver critically influences lipid metabolism. After being absorbed in the intestine, lipids are incorporated into chylomicrons and transported to peripheral tissues. The remaining fatty acids, estimated to comprise 5–30% of the original lipids, are imported into the liver and converted into triglycerides via combination with glycerol [93]. In hyperlipidemia, due to an excess of circulating lipids, the excess triglycerides accumulate in liver [94]. Excessive storage of triglycerides in hepatocytes leads to steatosis, followed by hepatocyte death [94]. The presence of dead cell debris induces neutrophil infiltration, a hallmark of hepatic inflammation [95]. Transforming growth factor (TGF)-β enforces the transformation of hepatic satellite cells into myofibroblasts, and platelet-derived growth factor (PDGF) augments myofibroblasts proliferation [96]. Myofibroblasts secrete substantial quantities of extracellular matrices, including α-smooth muscle actin and collagen, leading to fibrosis [96]. The resulting aggravation of liver fibrosis and inflammation eventually progress to cirrhosis and occasionally lead to hepatocellular carcinoma [97].

Based on the recent international criteria, steatotic liver disease (SLD) can be classified depending on the presence or absence of cardiometabolic risk factors [98,99]. MASLD refers to SLD without excessive alcohol consumption (where the threshold is >20 g/d for women and >30 g/d for men) [98]. Although diagnosis of NAFLD only focuses on alcohol intake, MASLD is diagnosed based on other metabolic disorders such as obesity, T2DM, high blood pressure, lipidemia, and hypo-high-density lipoprotein (HDL) cholesterolemia [100,101]. MASLD reciprocally influences other metabolic diseases such as T2DM, cardiovascular disease, and chronic kidney disease [102]. Severe MASLD with local inflammation, hepatocyte ballooning, and fibrosis is known as metabolic dysfunction-associated steatohepatitis (MASH), formerly called nonalcoholic steatohepatitis (NASH) [99].

Recent papers demonstrate that hepatic USP2b is responsible for the progression of MASLD. In mice, the consumption of water containing 23.1 g/L d-fructose and 18.9 g/L d-glucose for 20 weeks resulted in MASLD, manifesting in increased liver weight, steatosis, and hepatic inflammation [37]. Mice given abundant fructose showed substantially elevated USP2b levels in hepatocytes [37]. *Usp2* (isoform not specified) overexpression increased the accumulation of intracellular lipid droplets as well as pro-inflammatory cytokine production in cultured hepatocytes, while *Usp2* knockdown had the opposite effect [37]. Stimulation with fructose also increased 11β-HSD 1 and C/EBPα levels in hepatocytes and in the livers of mice [37]. Similarly to T2DM, it is plausible that the C/EBPα-cortisol axis contributes to USP2-induced lipid accumulation in hepatocytes. However, there is currently no direct evidence demonstrating that the USP2b-C/EBPα-cortisol axis actually aggravates MASLD. Further studies are required to verify the impact of the C/EBPα on USP2b-mediated MASLD pathology in vivo.

The involvement of hepatic USP2b in the pathogenesis of MASLD has recently been examined from a different point of view [38]. As described in Section 3.1, screening GEO datasets revealed that *USP2* mRNA (isoform not specified) was upregulated in patients with MASLD [38]. USP2b levels were remarkably elevated in the liver of HFD-fed MASLD model mice [38]. In liver-selective *Usp2*KO mice, body weight, liver weight, hepatic and serum triglycerides, and blood alanine aminotransferase (ALT) were slightly reduced after HFD feeding [38]. After HFD feeding, the livers of *Usp2*KO mice exhibited less infiltration of F4/80^+^ macrophages, elevated expression of an anti-inflammatory cytokines (*Il4*), and reduced expression of pro-inflammatory cytokines (*Tnf*, *Il1b*, and *Il6*), suggesting that USP2b excavates local inflammation in the liver [38].

Based on further in vitro and in vivo analysis, USP2 increased the expression of PPARγ and of its downstream genes in hepatocytes [38]. Mechanistically, overexpressed *USP2* (presumably USP2a) directly digested the K48-linked polyubiquitination chain at the K161 site of PPARγ in HEK293 cells, thus increasing the stability of PPARγ [38]. In cultured mouse hepatocytes, gene manipulation of *Pparg* reversed changes in triglyceride content by the overexpression or knockdown of the *Usp2* (isoform not specified) gene [38]. Therefore, PPARγ is likely to mediate the adverse effects on USP2 on MASLD. Coincidently, treating HFD-induced MASLD model mice with hepatocyte-targeting N-galactosamine-conjugated *Usp2* shRNA slightly but significantly moderated their MASLD [38]. This observation suggests that the specific targeting of hepatic USP2 might be applicable for treating MASLD in humans. Based on evidence that blockading USP2 reduces lipid droplet accumulation in hepatocytes in vivo (predominantly affecting USP2b) and in vitro (predominantly affecting USP2a), the authors suggest that both USP2a and USP2b play modulatory roles in the progression of hepatic lipidosis [38].

A more recent report demonstrated that USP2 (isoform not specified) promotes the differentiation of hepatic stellate cells into myofibroblasts by stabilizing p300 [103]. Together with the observation that the aberrant activation of USP2 isoforms contributes to hepatocellular carcinoma [54,104,105], these findings suggest that USP2 may act as an aggravating factor in the pathological progression from MASLD to hepatocellular carcinoma.

The liver plays pivotal roles in lipid metabolism [106]. Like other tissues, it generates ATP through β-oxidation [106]. In addition, the liver contributes to the synthesis of fatty acids, glycolipids, ketone bodies, and cholesterol [106]. Among them, approximately 80% of the de novo cholesterol is synthesized in the liver [107]. The remaining cholesterol derived from food ends up in the liver [108]. In addition to secreting cholesterol into the bile, the liver releases cholesterol and triglycerides into the blood as very-low-density lipoproteins (VLDLs) [94,109]. Cholesterol-rich low-density lipoprotein (LDL) is formed via the gradually removal of triglycerides in peripheral tissues [110]. The peripheral cells obtain cholesterol from LDL, while the remaining cholesterol is taken up in the liver by LDL receptors (LDLRs) [110].

Hepatic USP2 is also proposed to regulate lipid metabolism. Because USP2b has been found to localize predominantly in the peroxisome of cultured hepatocytes, it has been anticipated to regulate fatty acid β-oxidation [91]. USP2 has also been suggested to participate in cholesterol metabolism. Apolipoprotein O (ApoO) is thought to facilitate cholesterol efflux in mouse macrophage-like cells [111]. Microarray analysis of HepG2 cells reveals a negative correlation between *APOO* and *USP2* (isoform not specified) expression, supporting the regulatory role of USP2 in cholesterol metabolism in hepatocytes [112]. Consistent with this, USP2 was found to have the potential to influence cholesterol disposal by hepatocytes [39]. Inducible degrader of LDLR (IDOL) is an E3 ubiquitin ligase that ubiquitinates the intracellular region of LDLRs, leading to their internalization and subsequent digestion by lysosomes [113]. IDOL interacts with the catalytic C-terminal regions of USP2a and USP2b, and its stability is positively regulated by USP2a [39]. Paradoxically, further cellular analyses reveal that USP2 (isoform not specified) interferes with IDOL-elicited LDLR degradation [39]. Overexpressed USP2a is included in the complex encompassing LDLR and IDOL on the plasma membrane, and it interrupts the IDOL-dependent internalization of LDLRs [39]. Collectively, USP2 (or at least USP2a) appears to maintain LDL uptake by LDLRs in cultured hepatocytes.

### 3.3. USP2 in Skeletal Muscle

**Figure 3:**
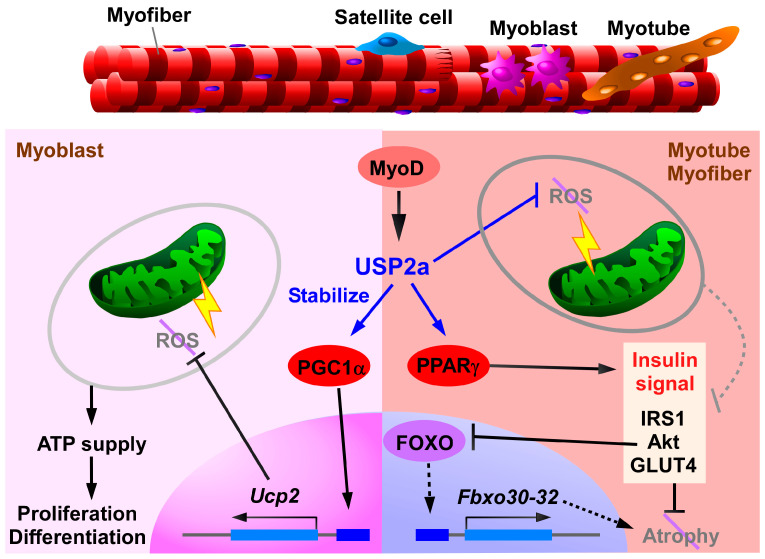
Putative roles of USP2a in skeletal muscle lineage on energy metabolism. (**Top**): Muscle cell lineage. Satellite cells are progenitor cells of muscle cell lineage and transform to proliferative myoblasts. Afterward, myoblasts fuse to form myotube and eventually become myofiber. (**Left bottom**): In cultured myoblasts, USP2 attenuated mitochondrial oxidative stress by sustaining the PGC1α–UCP2 axis, yielding ATP for proliferation and differentiation. (**Right bottom**): Anti-oxidative roles of USP2 are also observed in mature skeletal muscle (myotube and myofiber). Muscular USP2 stabilizes PPARγ, which sustains insulin sensitivity in prodiabetic conditions. Improved insulin signaling leads to the efficient usage of glucose in skeletal muscle and attenuation of FOXO-dependent induction of atrophy-related genes, including *Fbxo30*, *Fbxo31*, and *Fbxo32*. In this way, USP2 can prevent diabetic sarcopenia. This scheme was illustrated based on findings of previous studies [40,41,42,43].

In healthy humans, skeletal muscle accounts for 21–30% of body weight [114], therefore substantial affecting systemic energy metabolism. Since skeletal muscle is responsible for heat production and movement, it uses 70–90% of the glucose obtained from diet [115]. Muscle dysfunction therefore impedes systemic metabolism and subsequently accelerates a vicious cycle of metabolic diseases [116]. In elderly people, for instance, the skeletal muscle produces considerable quantities of myostatin, which hampers insulin signaling [117]. In contrast, irisin—which is secreted by skeletal muscle following exercise—reduced circulating triglycerides, total cholesterol, and LDL, and improved insulin sensitivity in aged mice [118]. In a study analyzing 63,330 patients across 2984 studies, sarcopenia was observed in ~24% of patients with MASLD, and was especially frequent in those with poor prognosis for MASLD [119]. Conversely, metabolic imbalance leads to muscle atrophy. Overnutrition causes hyperglycemia, lipid spillover by adipocytes, and bacterial translocation by a leaky gut, with myocellular repercussions [120]. USP2a has been reported to maintain muscular integrity, leading to the mitigation of metabolic disorders [40,41,42,43]. Based on various lines of evidence, USP2a in skeletal muscle is responsible for energy metabolism at the cellular, organ, and individual levels (Figure 3).

USP2a is relatively abundant in skeletal muscle [43,44,61]. *Usp2a* expression is directly upregulated by MyoD, a key transcription factor for skeletal myogenesis [43]. An earlier study demonstrated that the USP2a and USP2b have distinct roles in the differentiation of chicken muscle cells: USP2a positively regulates myofiber fusion, while the USP2b negatively regulates it [60]. Mechanisms underlying USP2a-participating myogenetic differentiation have been further analyzed using mouse-derived C2C12 myoblasts [40,41]. Gene KO and chemical blockade of USP2 reduced mitochondrial membrane potential, oxygen consumption, and the intracellular ATP level, along with a lack of differentiation ability; therefore, the importance of USP2 in myoblasts seems to be related to the supply of ATP by OXPHOS, which is necessary for myocyte differentiation [40]. Concurrently, USP2 depletion elicited a remarkable accumulation of reactive oxygen species (ROS) in mitochondria and reduced uncoupling protein (UCP) 2 levels [41]. Given that UCP2 attenuates the production of mitochondrial ROS [121,122,123], USP2 likely protects against ROS-elicited mitochondrial damage by inducing UCP2. In C2C12 cells, the introduction of USP2a prolongs the half-life of PGC1α by reversing K48-linked polyubiquitination [41]. Since PGC1α elevates *Ucp2* expression in C2C12 cells [124], USP2a confers anti-oxidative capacity by activating the PGC1α–UCP2 axis. Despite these observations, antioxidative roles of USP2a in myoblasts have been evaluated only using cellular models. Notably, mice with global or muscle-selective *Usp2*KO in grow normally, with no effects on body weight [43,48]; hence, the regulatory function of USP2 might only be exerted in deteriorative situations such as during the regeneration of muscle damage.

In addition to its effects on myoblasts, USP2 (isoform not specified) also attenuates mitochondrial oxidative stress in mature myocytes. In differentiated C2C12 cells, ML364 treatment increased mitochondrial ROS levels [42]. The chemical inhibition of USP2 disrupts mitochondrial complexes, thereby reducing mitochondrial membrane potential and intracellular ATP levels [42]. Moreover, diabetic *Usp2*KO mice frequently display malformed mitochondria accompanied by an accumulation of oxidative stress [42]. These findings suggest that muscular USP2 helps to prevent ROS-induced mitochondrial damage. It should be noted, however, that the protective effects of USP2 against oxidative stress in muscle were observed only under diabetic conditions, which are characterized by enhanced oxidative stress [125].

The beneficial roles of muscular USP2 in T2DM have been reported [42,43]. A microarray dataset (GSE156249) revealed that *USP2* mRNA (isoform not specified) was reduced in diabetic patients [43]. Similarly, diabetes model mice, which were induced by HFD-feeding and streptozotocin injection, exhibited reduced USP2a [43]. Although global or skeletal muscle-selective *Usp2*KO mice did not exhibit any abnormality in body weight and blood glucose levels, both KO types exhibited aggravated insulin resistance and glucose intolerance under diabetic conditions [42,43]. Consistent with this, *Usp2*KO reduced insulin receptor substrate 1 (IRS1) and glucose transporter 4 (GLUT4) levels in diabetic mice, while its overexpression increased these levels [43]. Therefore, muscular USP2, presumably USP2a, prevents progression of T2DM.

One possible mechanism underlying the anti-diabetic effects of USP2a is the mitigation of oxidative stress in skeletal muscle. Numerous studies have demonstrated that ROS impair insulin signaling by affecting multiple targets in muscle tissue [126,127,128]. Muscle-selective *Usp2*KO mice with severe T2DM exhibited remarkably elevated oxidative stress in the soleus muscle compared to control mice, suggesting that the antioxidative function of USP2a helps to maintain insulin sensitivity in skeletal muscle [42].

Another anti-diabetic effect of USP2a has been proposed to arise from its ability to mitigate muscle atrophy [43]. Diabetic *Usp2*KO mice show enhanced expression of muscle ring finger-1 (MURF-1) [43], a ubiquitin ligase that principally participates in muscle atrophy [129]. In the same diabetic mice, *Usp2* depletion also causes weight loss in the gastrocnemius, tibialis anterior, and soleus muscles, along with a decline in grip strength and running ability. On the contrary, the overexpression of *Usp2a* mitigates high blood glucose and insulin levels, an increase in muscular MURF-1, the loss of muscle mass, and defective muscle performance [43]. This provides evidence that USP2 sufficiently alleviates muscle atrophy and subsequent diabetes by mitigating metabolic disorders.

The molecular mechanisms by which USP2a represses muscle atrophy and consequent insulin resistance has also been investigated. USP2a stabilizes PPARγ by deubiquitinating the K184 and K185 on its surface. Consistent with this, PPARγ protein levels closely correlate with *Usp2* expression in diabetic mice and C2C12 myotubes treated with dexamethasone or TNF-α [43]. Notably, the USP2–PPARγ axis suppresses forkhead box subgroup O (FoxO)-driven expression of muscle-atrophy-related genes, including *Fbxo30*, *Fbxo31*, and *Fbxo32* [43]. Because the inhibition of PPARγ abolishes the beneficial effects of USP2a on muscle atrophy and insulin resistance, targeting the USP2a–PPARγ axis in muscle may be effective for treating both T2DM and sarcopenia.

### 3.4. USP2 in Adipose Tissue

**Figure 4:**
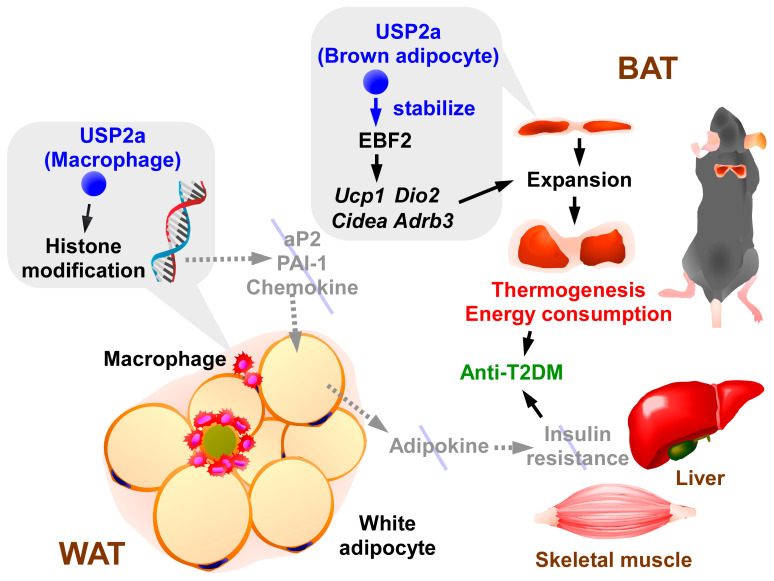
Putative roles of USP2a in adipose tissue macrophages and brown adipocytes. In WAT, macrophage USP2a modulates histone modification of T2DM-related genes, thereby reducing the expression of detrimental humoral factors such as aP2, PAI-1, and various chemokines. As a consequence, the secretion of adverse adipokines from adipocytes is attenuated. In brown adipocytes of BAT, USP2 stabilizes the transcription factor EBF2, which upregulates the genes responsible for BAT expansion such as *Ucp1*, *Dio2*, *Cidea*, and *Adrb3*, thus accelerating thermogenesis and subsequent energy consumption. Collectively, the USP2 in adipose tissue macrophages and brown adipocytes serves to prevent T2DM. This scheme was illustrated based on the findings reported in previous studies [44,46].

To date, the roles of USP2 in adipose tissues have been documented in several articles (Figure 4).

#### 3.4.1. USP2 in White Adipocytes

Adipose tissue, which is distributed throughout the body, primarily consists of adipocytes. The excessive accumulation of lipids in this tissue leads to obesity, the basis for metabolic syndrome. Adipocytes can be roughly classified into white, brown, and beige types. White adipocytes store excess energy as triglycerides in the cytoplasm. Overnutrition causes adipose tissue to expand through the hyperplasia (an increase in cell number) and hypertrophy (an increase in cell size) of white adipocytes [130,131,132]. Hypertrophic white adipocytes are less sensitive to insulin and trigger adipose tissue inflammation [130,131,133]. White adipocytes make up the white adipose tissue (WAT) in visceral and subcutaneous regions. The mass of visceral adipose tissue is closely related to metabolic disorders; in mice, the transplantation of subcutaneous adipose tissue has beneficial effects on energy metabolism [134,135].

The phytochemical Indol-3-carbinol (I3C) is enriched in cruciferous vegetables such as cabbage, broccoli, and mustards [136]. Intake of 3,3′-diindolylmethane (DIM), a metabolite of I3C, for 3 months significantly repressed body weight gain in HFD-fed mice [137]. Mouse-derived 3T3-L1 cells revealed that DIM inhibited adipogenic differentiation and caused the defective expression of mature adipocyte markers such as PPARγ, C/EBPα, adipocyte protein 2 [aP2, also called fatty acid-binding protein 4 (FABP4)], and FASN [137]. Furthermore, DIM-elicited defects in adipocyte differentiation could be attributed to cyclin D1 dysregulation during the early stages of differentiation [137]. On the other hand, DIM markedly inhibits the protease activity of USP2 [137]. Given that USP2 (isoform not specified) prevents the proteasome-dependent degradation of cyclin D1 [80], the authors speculated that USP2 might enhance adipogenesis through the stabilization of cyclin D [137]. However, this remains speculative, as no direct evidence demonstrates the role of USP2 in white adipocyte differentiation.

A recent study presents findings opposite to those described above [46]. Three weeks of the chemical inhibition of USP2 increased inguinal and epididymal fat mass in C57BL/6J mice and promoted adipocyte hypertrophy [46]. Similarly, local administration of *Usp2* shRNA into adipose tissue significantly increased fat mass [46]. These results suggest that USP2 is dispensable for the regulation of hypertrophy in WAT.

#### 3.4.2. USP2 in Brown and Beige/Brite Adipocytes

In contrast to white adipocytes, brown and beige adipocytes consume intracellular triglycerides, generating heat [138]. Stimulation of brown and beige adipocytes is therefore linked to recovery from metabolic diseases. Brown and beige adipocytes exclusively express UCP1, and this contributes to non-shivering thermogenesis [138]. In rodents, brown adipocytes are predominantly distributed in brown adipose tissue (BAT), which mostly occurs in the interscapular area [139,140] and is markedly innervated by sympathetic nerves [141]. A positron emission tomography (PET) study of mice revealed that BAT is also present in other parts of the body, showing topological similarity to thermogenic fat pads in humans [142]. Although brown adipocytes are always present in BAT after birth, “beige” (brown-like) or “brite” (brown-in-white) adipocytes emerge in subcutaneous adipose tissues under sustained activation of β3 adrenoreceptors [143].

Analysis of comprehensive GEO gene expression datasets reveals that *Usp2* (isoform not specified) is upregulated during active thermogenesis in the BAT of mice [46]. Similarly, inducing the browning of white adipocytes increased the expression of *Usp2* [46]. By contrast, *Usp2* was the most downregulated DUBs in the BAT of HFD-fed obese mice [46]. The USP2a protein level in the BAT and inguinal WAT was closely associated with UCP1 protein level in mice, implying that USP2a may be pivotal for the development of brown/beige adipocytes [46]. BAT-specific *Usp2* knockdown for 3 weeks reduced the volume of BAT and oxygen consumption in mice at ambient temperature [46]. Likewise, the systemic blockage of USP2 for 3 days also decreased the mass of BAT [46]. On the contrary, BAT-specific overexpression of *Usp2a* continuously increased UCP1 expression and oxygen consumption in obese mice [46]. These results collectively suggest that USP2a is essential for the formation of brown/beige adipocytes at a steady state. Remarkably, *Usp2a* overexpression in BAT suppressed body weight gain and improved insulin sensitivity and glucose tolerance in obese mice. These results indicate the therapeutic role of USP2a in BAT in T2DM [46]. Further proteomics analysis reveals that USP2 stabilizes early B cell factor (EBF2) by digesting K63-linked polyubiquitination [46]. EBF2 participates specifically in the differentiation of brown adipocytes in the dermomyotome [144]. Accordingly, *Ebf2* overexpression reversed the reduction in energy expenditure resulting from BAT-selective *Usp2* knockdown in mice [46]. Therefore, USP2a likely maintains EBF2 levels, thus enhancing brown adipocyte differentiation.

#### 3.4.3. USP2 in Adipose Tissue Macrophage

In obesity, adipose tissue exhibits low-grade inflammation. The rapid and excessive expansion of adipose tissue causes the hypoxia and cellular senescence of adipocytes [133,145]. Senescent adipocytes exhibit the senescence-associated secretory phenotype (SASP) and release pro-inflammatory cytokines [133,145]. The production of damage-associated molecular patterns (DAMPs) and saturated fatty acids from dead adipocytes provoke adipose tissue inflammation via several mechanisms [145]. Macrophages are the predominant inflammatory cell type involved in adipose tissue inflammation [145,146]. Macrophages infiltrate and surrounded the dead adipocytes, where they form a crown-like structure and secrete inflammatory cytokines such as TNF-α and IL-6 [133,145,146], which are both postulated to cause insulin resistance [147,148]. Recent studies identified novel subsets of macrophages associated with adipose tissue inflammation [146]. For instance, metabolically activated adipose-tissue macrophages (MMes) and CD9^+^ macrophages are thought to promote inflammation, leading to insulin resistance [149,150]. Furthermore, it has been argued that the impact of spillover products from adipose tissue on systemic metabolic disorders should be more precisely estimated [133].

The leptin deficiency in *ob*/*ob* mice results in severe obesity, leading to T2DM [151]. Compared to C57BL/6 mice, *ob*/*ob* mice exhibit a lower expression of *Usp2a* in F4/80^+^CD11b^+^ macrophages in their visceral adipose tissue, along with a higher expression of *Fabp4*, high-mobility group AT-hook 2 (*Hmga2*), and plasminogen activator inhibitor-1 (PAI-1, gene name *Serpin1*) [44], all of which are potentially involved in local inflammation and diabetes [152,153,154]. Accordingly, *USP2*-knockdown human macrophage-like HL60 cells exhibited enhanced the production of aP2, HMGA2, PAI-1, and several chemokines, including C-C motif chemokine ligand (CCL)-2, CCL-7, and CCL-24, while the reintroduction of USP2a suppressed the increments [44]. The therapeutic effects of *USP2*-overexpression on obesity and diabetes became evident in macrophage-selective *Usp2a* transgenic mice only after one year of HFD feeding, suggesting that USP2a present in macrophages has a marginal impact on metabolic disorders at the individual level. At that time point, the muscle and liver exhibited greater insulin sensitivity than the control C57BL/6 mice [45]. Although the specific molecular mechanisms remain unclear, the accessibility of chromatin to inflammatory molecules increased within *USP2*-knockdown HL-60 cells [44]. Together, these findings indicate that USP2a in adipose tissue macrophages slightly attenuate the progression of T2DM by modulating adipocyte secretion.

### 3.5. USP2 in Hypothalamic Neurons

Approximately sixty years ago, examining the electricity-induced lesions of hypothalamic nuclei identified the ventromedial hypothalamus (VMH) and lateral hypothalamus (LH) as the satiety and feeding centers, respectively [155]. In contrast, several hypothalamic nuclei, including the paraventricular nucleus (PVN) and arcuate nucleus (ARC), release hypothalamic hormones, some of which stimulate glucoregulatory pituitary hormones such as adrenocorticotropic hormone and growth hormone [156,157]. Hypothalamic nuclei also modulate the tone in sympathetic activation. For instance, VMH stimulates glycogenolysis in the liver [158] and thermogenesis in brown adipose tissue [159], both of which are mediated by sympathetic activation [160,161]. Modulating hypothalamic neurons therefore alters systemic energy metabolism.

**Figure 5:**
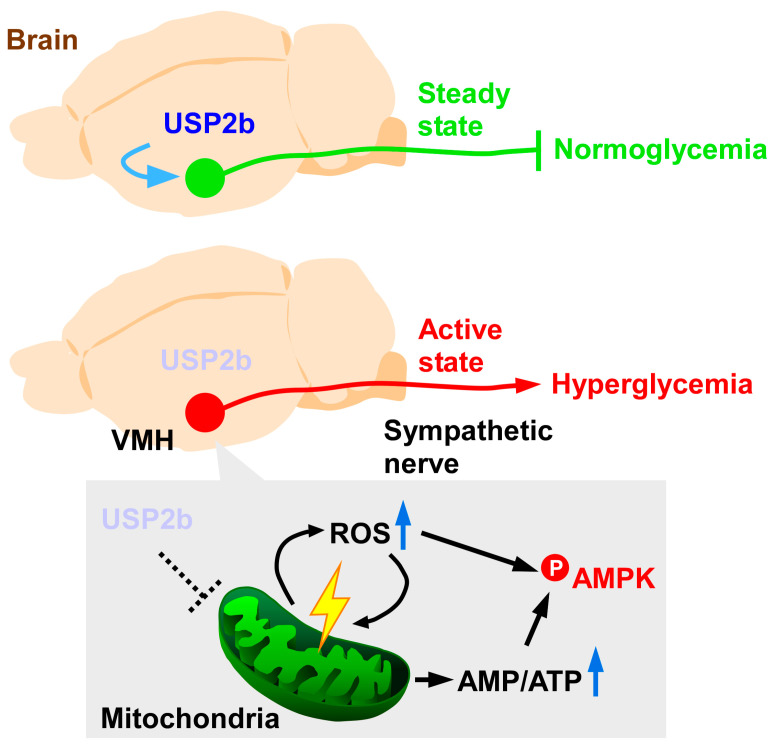
Putative roles of hypothalamic USP2b in blood glucose control. (**Top**): USP2b in the VMH suppresses excessive sympathetic nerve activity, thereby normalizing sympathetic tone. Middle: Inhibition of USP2b leads to sympathetic malactivation, resulting in hyperglycemia. (**Bottom**): Hypothetical molecular cascade in VMH neurons. Impaired USP2b function causes accumulation of mitochondrial ROS, which directly and indirectly promotes AMPK phosphorylation. This activation of AMPK triggers sympathetic nervous system activation. This scheme is illustrated based on the findings reported in a previous study [47].

Previous reports indicate the involvement of hypothalamic USP2 in systemic glucose metabolism (Figure 5). Based on microarray and subsequent quantitative PCR (qPCR) analyses, starvation and insulin-induced severe hypoglycemia evoke a significant increase in cerebral *Usp2c* expression in the hypothalamus and cerebral cortex of mice, indicating the regulatory roles of USP2 in energy metabolism [162]. Histological data from mice indicates that *Usp2b* mRNA is only abundant in neural cells in hypothalamic nuclei such as the VMH, LH, ARC, PVN, and the dorsomedial hypothalamus [47]. In mice, the administration of ML364 only to the VMH increased circulating norepinephrine levels and subsequently increased blood glucose [47]. Since intra-VMH injection of ML364 substantially promotes hepatic glycogenolysis, USP2 in the VMH seems to mitigate hyperglycemia by inhibiting sympathetic nerve-dependent glycogenolysis [47]. ML364 treatment promotes the accumulation of mitochondrial ROS in neural cells in vitro and in vivo, thereby negatively impacting mitochondrial ATP synthesis; the resulting ATP depletion in the hypothalamic neurons activates AMP-activated protein kinase (AMPK) [47], which elicits hepatic sympathetic activation [163,164]. Pretreatment of the VMH with AMPK inhibitor compound C or Trolox (a ROS scavenger) rescued the ML364-elicited increases in serum norepinephrine, hepatic glycogen phosphorylase activity, and blood glucose [47]. These findings indicate that the USP2 (presumably USP2b) in VMH neurons controls blood glucose in an ROS–AMPK-dependent manner. Because intra-VMH administration of ML364 produced clear effects even in lean mice, hypothalamic USP2b may suppress excessive sympathetic activation, thereby helping to maintain blood glucose at a normal level.

It should be noted that our preliminary histochemical analysis using probes for *Usp2a* mRNA also detected a weak signal in hypothalamic neurons. Further studies are therefore needed to determine whether specifically USP2b is responsible for regulating blood glucose level. In addition, ML364 is known to inhibit USP8 [80]. Given that USP8 can influence brain function [165] and mitochondrial activity [166], genetic approaches will be required to clarify the specific contribution of hypothalamic USP2 to blood glucose regulation.

### 3.6. USP2 in Vascular Macrophages

Aberrant lipid metabolism causes circulating LDL to increase. LDL occasionally undergoes oxidization to form oxidized LDL (oxLDL), which is incorporated into macrophages by scavenger receptors such as CD36, lectin-like oxidized LDL receptor-1 (LOX-1), and macrophage scavenger receptor 1 (MSR1) [167]. Lipid-laden macrophages, known as “foam cells,” accumulate in the arterial intima, forming an atherosclerotic core with smooth muscle cells and other immune cells [168]. In this process, foam cells provoke local inflammation and ferroptosis [169,170], and aggregated dead foam cells form an atherosclerotic core and lead to intimal thinning by causing local tissue remodeling [171]. Atherosclerosis is closely related to cardiovascular diseases, which are a major cause of mortality. In 2021, cardiovascular diseases such as stroke and heart attack caused ~6.2 million deaths in the Western Pacific region, accounting for ~40% of deaths globally [172].

To analyze atherosclerosis, mice and rats develop almost no atherosclerotic lesions, even when fed a HFD or high-cholesterol diet. Thus, genetic manipulation is required to facilitate the generation of atherosclerosis in mice and rats. Double or single disruptions of the apolipoprotein E (*Apoe*) and *Ldlr* genes, along with feeding a high-cholesterol diet, are widely used to induce atherosclerosis mouse models [173,174]. Double knockout of both *Apoe* and *Ldlr* brings about detectable plaques in the aorta by 15 weeks [175]. Intravenous injection of an adeno-associated virus (AAV)-encoding proprotein convertase subtilisin/kexin type 9 (PCSK9) in combination with HFD for 20 weeks causes severe atherosclerosis and T2DM [176].

As aforementioned, USP2a maintains surface expression of LDLR and enhances LDL uptake in cultured human hepatocytes [39]. Because *Usp2*/*USP2* knockdown also reduces LDL uptake in HeLa cells and decreases LDLR protein levels in mouse embryonic stem cells [39], USP2a appears to promote LDL uptake in several cell types, including macrophages. However, macrophages primarily uptake LDL through scavenger receptors rather than LDLRs [167]. Therefore, the IDOL–LDLR axis in macrophages is unlikely to play a major role in atherosclerotic plaque formation.

The potential involvement of macrophage USP2 in the pathogenesis of atherosclerosis has been revealed by examining the effects of *Panax notoginseng* saponins (PNS), a plant extract. PNS significantly reduce the number and area of atherosclerotic plaques in the aorta of 16 weeks-HFD-fed *Apoe*KO mice [50]. Within these lesions, PNS inhibit the lipid accumulation, inflammatory activation, and ferroptosis of macrophages. Notably, PNS markedly downregulate the expression of *Usp2* (isoform not specified), but not *Usp7*, *Usp9x*, *Dub3*, or *Usp15* in peritoneal macrophages from *Apoe*KO mice [50]. Because USP2 depletion abolishes the beneficial effects of PNS on ferroptosis and foam-cell formation, PNS may prevent atherosclerosis through downregulation of *Usp2* [50].

Peritoneal macrophages from HFD-fed *Usp2*KO mice displayed reduced lipid accumulation and decreased *Il1b* expression, along with lower serum IL-1β and IL-6 levels [50]. In addition, *Usp2*KO increased transcripts of glutathione peroxidase 4 (GPX4) and ferritin heavy chain, while reducing transcripts of acyl-CoA synthetase long-chain family member 4 (ACSL4) and malondialdehyde content [50]. These changes indicate that USP2 inhibition suppresses ferroptosis in macrophages. Mechanistically, USP2a, while it is reported to have an apparent molecular weight of ~110 kDa, deubiquitinates Kelch-like ECH-associated protein 1 (Keap1) in macrophages [50]. As Keap1 retains nuclear factor E2-related factor 2 (NRF2) in the cytoplasm, USP2a appears to inhibit NRF2 nuclear translocation by stabilizing Keap1 [177]. Since NRF2 is a central transcriptional regulator of the anti-ferroptosis pathway [178], USP2a likely promotes ferroptosis by stabilizing the Keap1-NRF2 complex in macrophages [50]. Accordingly, macrophages from *Usp2*KO mice display increased GPX4 and iron-storage protein levels, enhanced nuclear NRF2 accumulation, and reduced atherosclerotic plaque formation [50]. Collectively, macrophage USP2a disrupts NFR2 nuclear translocation, thereby creating conditions that favor the development of atherosclerosis.

### 3.7. USP2 in Testes

Infertility is internationally defined as a condition in which a couple is unable to conceive following unprotected intercourse for one year [179]. Approximately half of infertility cases are attributed to male sexual dysfunction [180], and asthenozoospermia (defective sperm motility) occurs in 82% of infertile men [181]. Sperm cells are capable of significant movement using their flagellae, suggesting that sperm exhibit high ATP demand. As with other cell types, sperm cells obtain ATP via both the anaerobic pathway (glycolysis) and aerobic pathway (OXPHOS). There is ongoing debate about whether OXPHOS or glycolysis is the primary source of ATP in sperm [182], and the dependence of sperm on the ATP supply systems varies among species [183]. Over long periods, glycolysis is believed to be the predominant source of ATP in mouse spermatozoa, and the pivotal roles of mitochondria in ATP production in mouse sperm have been revealed [183].

**Figure 6:**
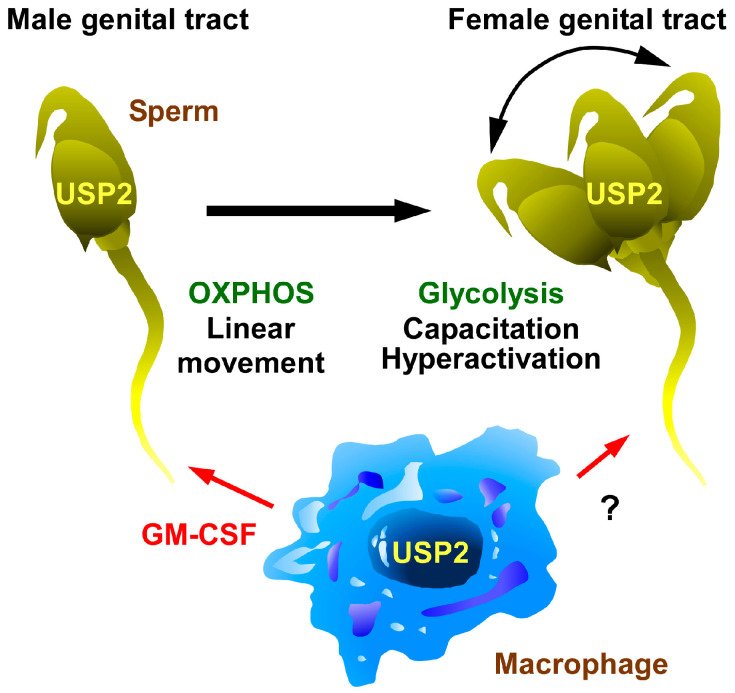
Putative roles of USP2 in sperm activity based on findings of previous studies [48,49]. In sperm cells or spermatids, USP2 is required to generate energy for motility and hyperactivation using nutrients supplied from body fluids. In macrophages located within the reproductive tract, USP2 supports simple linear movement, capacitation, hyperactivation, and asymmetric motility of sperm. Linear movement is dominantly driven by ATP produced through OXPHOS, whereas the other motility patterns are mainly powered by glycolysis. Macrophage USP2 contributes to the production of GM-CSF, which enhances OXPHOS in sperm in a paracrine manner. At present, it remains unclear which USP2 isoform predominantly modulates fertilization process in sperm/spermatids and in macrophages.

Several studies have suggested the involvement of USP2 in energy metabolism in mouse sperm (Figure 6). *Usp2*-deficient mice exhibit severe male infertility, with the formation of multinucleated cells in the lumen of the seminiferous tubules [48]. Although the sperm of *Usp2*KO mice moved normally in culture media, they rapidly lost their motility in PBS [48]. Since PBS contains essential ions such as calcium, magnesium, and bicarbonate, the rapid loss of function of *Usp2*KO sperm appears to be due to the disruption of ATP supply. In the testes, USP2a and USP2b is remarkably expressed in the late stages of spermatids [56]. The expression level of either isoform of USP2 is likely to determine nutrient utilization in sperm.

Frozen-thawed mouse sperm from mice with myeloid-selective *Usp2*KO exhibits increased damage to mitochondrial OXPHOS and decreased simple linear motility. *Usp2*KO sperm also manifests suppressed hyperactivation and in vitro fertilization [49]. Testicular macrophages abundantly express granulocyte–macrophage colony-stimulating factor (GM-CSF) [49], which potentiates sperm activation [184]. Accordingly, myeloid-selective *Usp2*KO mice show reduced expression of *Csf2* (which encodes GM-CSF) in testicular macrophages [49]. Adding GM-CSF to freeze-thawed sperm from myeloid-selective *Usp2*KO mice restored OXPHOS, intracellular ATP levels, and linear motility, regardless of their reduced hyperactivation and inefficient capacitation [49]. Therefore, certain isoforms of USP2 in testicular macrophages indirectly protect mitochondrial ATP synthesis in sperm, resulting in the maintenance of non-hyperactivated mobility. When treating infertility in humans, conserving rare species, and managing livestock resources, the roles of USP2 in testicular macrophages may have important implications.

Since neither study employed isoform-specific *Usp2*KO models, we cannot specify which isoform is responsible for maintaining sperm activity. However, the relative contributions of sperm-derived versus macrophage-derived USP2 can be inferred. Myeloid-selective *Usp2* deficiency only affected freeze-thawed sperm [49], whereas global *Usp2*KO mice exhibited severe male infertility [48]. These findings suggest that macrophage USP2 has modulatory roles in supporting sperm activity, whereas USP2 in sperm (or spermatids) is necessary for successful fertilization.

### 3.8. USP2 in Cancerous Cells

Many studies on USP2 (mainly USP2a) have focused on its role in carcinogenesis. USP2 regulates the stability of carcinogenesis-associated molecules such as cyclin D1 [73,80], cyclin A1 [81], S-phase kinase-associated protein 2 (SKP2) [185], p53 [186], murine double minute 2 (Mdm2) [187], and Twist [188]. Further, USP2a reduces antitumor immunity by inducing programmed death-ligand 1 (PD-L1) [83]. USP2a regulates oncogenesis by targeting metabolic enzymes. Since cancer cells exhibit marked proliferation, they require very large amounts of energy to drive the related biochemical reactions. To support their excessive proliferation, they utilize FFAs (mainly palmitate) as fuel sources (via β-oxidation) and as sources of components for the plasma membrane [189]. Palmitate is synthesized from acetyl CoA by FASN, which is robustly expressed in malignant cancer cells [189,190]. Considerable evidence indicates that FASN is responsible for poor prognosis in various types of cancer [191,192,193,194,195]. In contrast, disturbance of FASN stops cancer progression [190,196]. In human prostate cancer LNCaP cells, the ubiquitin proteosome pathway determines the stability of FASN, and USP2a sustains FASN levels by removing the polyubiquitin chain. *USP2* silencing induces apoptosis by reducing FASN levels, while adding FASN overcame USP2a deficiency-induced apoptosis [52]. In glioma, USP2a and FASN mRNA and protein levels were similarly elevated, depending on the extent of the malignancy [53]. The role of USP2a in carcinogenesis has also been reported in hepatocellular carcinoma. USP2a is abundant in hepatocellular carcinoma cells, and a significant proportion of USP2a is directly associated with FASN in hepatocellular carcinoma cells from patients with poor prognosis [54]. RNA’s interference with *USP2* significantly reduces FASN protein levels, indicating that USP2a is detrimental to sustained FASN levels in hepatocellular carcinoma [54]. Overexpression of *AKT* results in hepatocellular carcinoma with lipogenesis in mice, leading the authors to conclude that aberrant activation of AKT is a prerequisite for hepatocellular carcinoma [54]. Importantly, AKT-induced preneoplastic and tumor lesions show elevated USP2a levels, which correlate with the tumor grade [54]. This suggests that the USP2a–FASN axis contributes to the progression of hepatocellular carcinoma.

Autophagy is a crucial catabolic process involved in FFA metabolism whereby neonates and starving animals obtain glucose and amino acids. For example, autophagy augments lipolysis for the production of FFAs, thus promoting OXPHOS for the differentiation of neutrophils [197]. Acute myeloid leukemia promotes autophagy to obtain FFAs to drive OXPHOS [198]. Conversely, FFAs also stimulate autophagy [199]. In osteosarcoma (OS), USP2a potentiates autophagy through FFA production and OS proliferates in an autophagy-dependent manner [51]. The formation of the autophagosome and autolysosome in OS depends on FASN, which is elevated during the activation of autophagy [51]. Based on cell culture experiments, FASN-induced autophagy is necessary for the proliferation, migration, and resistance to cisplatin [51]. Immunoprecipitation and mass spectrometry analysis reveal that FASN is associated with valosin-containing protein (VCP), a multifunctional AAA-ATPase [200]. VCP recruits USP2a to deubiquitinate FASN in OS [51]. Following subcutaneous xenograft or orthotopic transplantation of VCP-overexpressing OS cells into nude mice, the progression of tumor growth was observed; in contrast, gene silencing of *USP2* or *FASN* reduced the adverse effects of VCP in cancer cells [51]. Together, these results indicate that the USP2a–VCP–FASN complex critically supports malignancy in OS by inducing autophagy.

## 4. Current Technical Issues in USP2 Research Related to Energy Metabolism

Previous studies have provided substantial insights into the potential roles of USP2 in energy metabolism. However, it is also important to recognize that each experimental approach carries technical limitations that must be addressed. Particularly, the overestimation of the function of USP2 and insufficient clarity regarding its mechanisms of action hinder the development of USP2-target therapies. In this section, we summarize the key methodological issues that must be considered to appropriately interpret the roles and contributions of USP2 to energy metabolism and metabolic disorders.

### 4.1. Issues with Animal Models

Most current knowledge of the in vivo functions of USP2 derives from mouse models. However, as emphasized in recent discussions of new approach methodologies (NAMs), findings obtained in laboratory rodents do not always translate to humans [201,202]. Rodents and humans differ substantially in energy metabolism profiles; glucose metabolism, glucose disposal, and glycogen storage patterns vary extensively between the two species [203]. For example, whereas skeletal muscle consumes a large proportion of circulating glucose in humans, the liver accounts for a comparatively large share in mice [203]. Likewise, neural and muscular cells from mice, rats, and humans exhibit distinct gene expression programs [204,205]. In both species, pancreatic islets differ in ATP production and insulin secretion in response to glucose [206], consistent with species-specific islet transcriptomes [207,208]. Lipid metabolism also diverges: ~80% of circulating cholesterol is carried in HDL in mice, whereas ~75% reside in VLDL/LDL fractions in humans [209]. Moreover, because obesity is a polygenic, rodent monogenic models cannot fully recapitulate most human disease states [210]. Before applying animal data to clinical practice, it is therefore essential to bridge the human–mouse gap. In addition to NAMs, several strategies have been used to generate humanized animal models, such as organ or tissue transplantation or blastocyst complementation [211,212,213,214]. Because human cells largely retain their intrinsic properties in these contexts, studies using humanized mice may provide a more accurate understanding of the role of USP2 in human energy metabolism.

### 4.2. Issues with Cellular Models

Many studies have examined the function of USP2 using only cultured cells [40,41,82,91,215,216]. Although cellular and animal models often exhibit the same roles for USP2, findings observed in vitro are not always reproduced in vivo. For instance, although USP2 silencing interrupted myoblast proliferation and differentiation in cell culture [40,60], *Usp2*KO mice grew and developed normally [48], indicating that muscular USP2 does not hamper the organogenesis of skeletal muscle. Similarly, while *Usp2*KO mice displayed normal sodium balance [217], in vitro analysis reveals that USP2b regulates the surface expression of epithelial sodium channel [218]. Additionally, the microenvironment of cells in conventional culture systems differs substantially from that of cells in human tissues. In terms of energy metabolism, the cellular environment differs substantially between in vivo physiological conditions and conventional cell culture. For instance, while cultured cells are continuously exposed to atmospheric oxygen (~21%), the concentration of oxygen within tissues ranges from 2% to 9% [219]. The excessive levels of oxygen in cell culture expedite OXPHOS, thus yielding enough ATP for proliferating and producing mitochondrial ROS as a byproduct. As we proposed previously, if USP2 functions as a common mitigator of oxidative stress [40,41,42,47], its function in protecting ATP production will be more prominent in cultured cells than in animal tissues. Furthermore, cultured parenchymal cells lack the humoral and neural regulation present in vivo, as well as interactions with stromal cells such as immune cells. These cells are also grown without share stress under hydrodynamic conditions that differ substantially from those in the human body [220]. In this context, recent advances in microphysiological systems (MPSs) offer a promising approach to bridge the biological gap between conventional cell cultures and human physiology [220,221]. MPS platforms may therefore help us to clarify the roles of USP2 in human energy metabolism with greater physiological relevance.

### 4.3. Issues of Dominant USP2 Isoforms

RNA interference, gene KO, and pharmacological inhibition have been the gold standard approaches for assessing the roles of USP2 in energy metabolism in vitro and in vivo [36,42,43,46,47,222]. However, these approaches do not identify which USP2 isoform is responsible for the observed phenotypes. Although the relative expression levels of each isoform may provide a strong indication of the dominant variant in a given tissue, expression alone is not conclusive. Each USP2 isoform differs in its subcellular localization [44,56,61], which partially explains the isoform-specific functions observed across different cell types [57,58,60,223]. Although some studies utilized the overexpression of each isoform, isoform-specific KO models might be more effective for accurately verifying the roles each variant have in energy metabolism. Considering that the N-terminal extension of USP2a can bind target proteins [187] and determine substrate specificity [56], animal models lacking this extension may be useful for developing USP2a-targeted therapies and clarifying the isoform-specific biological roles of USP2 in metabolism.

### 4.4. Issues of Target-Selectivity in Genetic and Chemical Manipulation

To evaluate the systemic effects of USP2 inhibition, ML364 has been widely used as a USP2 inhibitor [46,47,103,224]. However, ML364 also significantly inhibits USP8 and USP48 [80,225]. Therefore, the phenotypes observed with ML364 should be interpreted as the combined consequence of selectively inhibiting USP2, USP8, and USP48 at a minimum. As recommended in previous reports [40,46], pairing ML364 treatment with genetic models (e.g., KO or KD) increases the credibility of the conclusions drawn from the chemical approach. In terms of the genetics, RNA interference-mediated gene knockdown and CRISPR/Cas9-mediated knockout can produce off-target effects [226,227]. Rigorous validation remains essential, although multiple strategies exist to mitigate off-target activity: confirming the results across independent cell clones or animal lines; using orthogonal guiding RNAs/small interfering RNAs (siRNAs); or including rescue experiments with siRNA-resistant cDNA or CRISPR-proof constructs.

Overexpression models are often used to elucidate events controlled by USP2, such as those generated using transgenic mice and recombinant virus infection [36,43,44,46]. However, the results obtained in this way occasionally lead to overestimation because ectopically expressed proteins sometimes exhibit unexpected localization and functions [228,229,230]. Considering that the localization of USP2 determines its opportunities to encounter substrates, the overexpression of *Usp2* may cause interactions with unanticipated targets. To approximate the endogenous levels more closely, tetracycline-dependent inducible systems can be employed to titrate expression toward physiological ranges [231]. As an additional internal control, studies that demonstrate reciprocal phenotypes provide stronger causal support, e.g., knockout/knockdown versus overexpression producing opposite outcomes [36,43,46].

### 4.5. Issues of Tissue-Selectivity in Genetic Manipulation

When investigating the regulatory roles of USP2 in specific tissues, tissue specificity is critical. Accordingly, the tropism of tissue-targeted gene delivery systems must be carefully validated, such as adenoviral vector [36], AAV serotypes [43,46,232], and N-acetylgalactosamine conjugation [38]. Combining these delivery systems with tissue-selective promoters can further enhance the specificity of USP2 modulation.

Because USP2 is relatively broadly expressed and not highly substrate-specific, rigorous evaluation of tissue-selective *Usp2*-manipulation is necessary to accurately define its functions. Conditional *Usp2* KO mice generated using cell-selective Cre-*loxP* systems also require careful interpretation. For example, Nestin-Cre is widely used to target neural and glial progenitors [233,234,235], but Cre activity is also detected in the kidney and testes in Nestin-Cre mice [236,237], necessitating caution when attributing phenotypes to the central nervous system alone. Similarly, LyzM-Cre, which is commonly used for myeloid-selective KOs [205,238,239], exhibits substantial Cre expression in neurons of the motor cortex, cerebellum, and hippocampus CA3 region [240]. These known patterns of off-target Cre activity underscore the importance of stringent controls and orthogonal strategies.

### 4.6. Issues with Experimental Condition in Animal Studies

Accurately interpreting the roles of USP2 in each tissue requires careful attention to experimental conditions. For the glucose metabolism in particular, it is essential to specify whether USP2 controls the metabolism under steady state versus disease conditions within a given tissue. It should also be recognized that animal disease models are developed for distinct purposes, and their pathophysiological features can differ substantially. For example, a commonly used T2DM model combines HFD feeding with low-dose streptozotocin administration [42,43,241,242,243]. Although this model induces hyperglycemia rapidly [42,244], it does not produce notable obesity prior to hyperglycemia [42,241]. Consequently, it is not appropriate to use for evaluating the roles of USP2 in obesity-induced diabetes. Additionally, several genetically induced diabetic models, such as *ob*/*ob* mice, *db*/*db* mice, and Zucker diabetic fatty rats, exhibit severe adiposity in the adolescent phase [245,246], suggesting that they are not fully appropriate for modeling pathologies associated with middle-age in humans. Understanding the strengths and limitations of each model will facilitate the rational design of experiments and the development of USP2-target therapies, ensuring that the chosen model aligns with the specific clinical context being modeled.

### 4.7. Compensation by Other Molecules

Potential compensation for USP2 deficiency must be considered when interpreting results from chronic *Usp2* knockdown and *Usp2*KO models. Because the ubiquitination state of a substrate reflects the balance between ubiquitin ligases and DUBs [27], both arms of this system can buffer the loss of USP2 and confound phenotype attribution. For example, USP28 digests polyubiquitin chains from PPARγ, similar to USP2 [34]. Given that the *USP28* gene is also downregulated in the skeletal muscle of patients with T2DM [43], USP2 and USP28 may corporately protect against diabetic muscle atrophy by stabilizing PPARγ. Likewise, paralleling the actions of USP2 in macrophages [50], overexpression of USP7 increases Keap1, suppresses NRF2 activation, and reduces intracellular ROS in osteoclasts [247]. Because USP7 aggravates atherosclerosis in *Apoe* KO mice [248], USP7 may share pro-atherogenic roles with USP2 in myeloid compartments. Compensation may also occur upstream and/or downstream of USP2 substrates (for instance, through changes in E3 ligase activity, alternative DUBs targeting the same substrate, or pathway-level rewiring), making it challenging to delineate the full modulatory scope of the role of USP2 in energy metabolism. To address these issues, future studies could leverage combinatorial and multiplex genome editing to create multi-USP deletions, thereby defining the minimal DUB sets required for the progression of metabolic diseases. Complementary approaches, such as acute isoform-specific perturbations and temporal control (e.g., inducible systems), will further clarify which effects are uniquely attributable to USP2.

## 5. Cross-Sectional View of USP2 Across Tissues

In addition to the technical issues discussed in Section 4, comparing the functions of USP2 across tissues is necessary to understand its precise roles at the organismal level. In this section, we reinterpret the apparently contradictory findings from recent studies and contextualize them by experimental conditions and tissue biology.

### 5.1. Diversities of Outcomes by USP2 in Different Tissues

#### 5.1.1. Glucose Metabolism

Differences in the function of USP2 across tissues are particularly evident in the regulation of glucose metabolism. To broadly summarize these differences, hepatic USP2 promotes hyperglycemia [36,38], while USP2 in the hypothalamus, skeletal muscle, BAT, and adipose-tissue macrophages contributes to lowering blood glucose levels [42,43,45,46,47]. Although a purely biological explanation for these opposing roles remains speculative, many of the apparent inconsistencies can be resolved by reconsidering the experimental context in which each finding was obtained.

A critical factor is whether the animals were examined under steady-state or diabetic conditions. In skeletal muscle and adipose tissue macrophages, the genetic manipulation of *USP2a* only worsens hyperglycemia and insulin resistance in diabetic settings, suggesting that USP2a in these tissues acts primarily as a modulator during metabolic stress rather than under normal physiology [42,43,45]. In contrast, USP2 in the liver, BAT, and ventromedial hypothalamus exerts regulatory effects even at a steady state, implying its physiological and pathological functions in these tissues [36,46,47].

Understanding the biological context of each tissue further clarifies these distinctions. In skeletal muscle, USP2a preserves muscle mass, which is an important determinant of systemic glucose disposal. The benefit that muscular USP2a has on glucose regulation therefore reflects its capacity to maintain myofiber integrity and prevent atrophy [43]. In brown and beige adipocytes, USP2a promotes differentiation and tissue remodeling (“browning”), so its metabolic benefits arise gradually through structural changes rather than immediate enzymatic regulation. This is consistent with observations that 3 weeks are required for improvements in BAT to manifest following USP2a manipulation [46]. In the hypothalamus, USP2 (presumably USP2b) restricts sympathetic overactivation in the VMH, thus restraining hyperglycemia through acute neuroregulatory pathways [47]. In adipose tissue macrophages, however, USP2a influences glucose metabolism by suppressing the transcription of inflammatory humoral factors that impair insulin sensitivity [44]. Finally, hepatic USP2b plays a direct and rapid role by modulating the expression of metabolic enzymes with diurnal variation, thereby driving gluconeogenesis and lipogenesis [36].

A whole-body view is obtained from global *Usp2*KO or systemic USP2 inhibition. Under T2DM conditions, global *Usp2*KO mice exhibit exacerbated insulin resistance, impaired glucose intolerance, and elevated fasting blood glucose and insulin [43], indicating that the collective action of USP2 across tissues is beneficial for glucose homeostasis. Therefore, USP2 improves glucose metabolism as a whole body. These observations suggest that systemic USP2 inhibition could offer therapeutic potential for T2DM, although possible tissue-specific adverse effects, particularly in the liver, must be carefully evaluated.

#### 5.1.2. MASLD Progression

Hepatic USP2b has been reported to worsen hepatic pathology in MASLD models [37,38]. However, MASLD is strongly shaped by inter-organ interactions, particularly between the liver and skeletal muscle [249,250]. In our recent study, muscle-selective *Usp2* deletion did not produce pathological changes in the liver or muscle in a MASLD context [222], suggesting that muscular USP2 alone does not significantly influence MASLD progression [222]. This conclusion, however, may depend on the model. We used choline-deficient L-amino acid-defined HFD, which reduces hepatic VLDL export due to choline deficiency (choline is required for phosphatidylcholine synthesis), producing steatosis without hyperglycemia [251,252]. In contrast, approximately 70% of patients with diabetes have MASLD [253]. Therefore, to evaluate whether muscular USP2 contributes to MASLD complications, it may be more appropriate to use models that develop hyperglycemia, as in prior hepatic USP2 studies [37,38].

#### 5.1.3. LDL Cholesterol Metabolism and Atherosclerosis

In human hepatocyte models, USP2a and USP2b can increase LDLR surface expression and enhance LDL uptake [39], implying a potential to lower circulating LDL. However, this has been only demonstrated in vitro [39]. In vivo, serum LDL did not differ between HFD-fed *Usp2*KO and wild-type mice in a recent report [50], suggesting that hepatic USP2 (possibly USP2b) is not a dominant determinant of circulating cholesterol in mice. In the same study, HFD-fed *Usp2*KO mice displayed fewer and smaller atherosclerotic plaques than controls [50], implicating macrophage USP2a as sufficient to aggravate atherosclerosis, independent of hepatic LDL control. Species differences are also relevant: LDL is a minor cholesterol carrier in mice [209]. Accordingly, to define the roles of hepatic USP2b in human-like LDL physiology and atherosclerosis, human-relevant models should be used. Gene-engineered rabbits, which exhibit a lipoprotein profile more similar to humans, may be particularly informative [254].

### 5.2. Common and Distinct USP2-Driven Molecular Events Across Tissues

#### 5.2.1. Responses to Oxidative Stress

From a cross-sectional perspective, USP2 isoforms control several common intracellular systems across tissues, leading to both local and systemic effects on energy metabolism. Similar to observations in cancer cells [255], our series of studies suggests that USP2 isoforms function as mitigators of mitochondrial oxidative stress in neurons and myocyte lineages [40,41,42,47]. Because mitochondrial ROS accumulation and mitochondrial dysfunction exacerbate each other [256], USP2 may interrupt this vicious cycle and thereby preserve mitochondrial integrity. Consistent with this notion, both our group and others have identified multiple USP2 molecular targets involved in mitochondrial maintenance and oxidative stress control [41,257]. For example, USP2 deubiquitinates mytofusin-2, which is an essential regulator of mitochondrial fusion, thereby protecting cardiomyocytes from oxidative stress [257]. Additionally, USP2a stabilizes PGC1α, a key transcriptional regulator of mitochondrial biogenesis and function [258]. We previously proposed a model in which the USP2a-PGC1α axis reduces mitochondrial ROS through the induction of UCP2 [41]. In contrast to these protective roles, USP2a appears to promote oxidative stress in macrophages. In this context, USP2a removes polyubiquitin chains from Keap1, thereby retaining NRF2 in the cytoplasm and leading to increased intracellular ROS accumulation [50]. Given that the PGC1α-NRF2 complex is known to support mitochondria biogenesis [259], the macrophage findings appear to contradict observations made in muscle and neuronal tissues. At present, the molecular basis for this tissue-specific divergence remains unclear. One possible explanation lies in subcellular localization: Keap1 is predominantly cytoplasmic and remains so even upon inhibition of nuclear export by leptomycin B [260], whereas PGC1α undergoes dynamic shuttling from the cytoplasm to the nucleus [261]. Because USP2a itself shuttles between nuclear and cytoplasmic compartments [44], it is plausible that USP2a associates with distinct scaffold proteins in different tissues, resulting in divergent subcellular localization and substrate accessibility. Further studies aiming to comprehensively identify USP2-interacting proteins in specific subcellular fractions will be essential to clarify these context-dependent effects.

#### 5.2.2. PPARγ-Modulated Events

Another major molecular pathway regulated by USP2 across tissues involves PPARγ. In skeletal muscle, USP2a ameliorates T2DM by stabilizing and activating PPARγ, which in turn suppresses muscle atrophy and improves insulin sensitivity [43]. In this tissue, PPARγ protects against angiotensin II-induced muscle atrophy by reducing mitochondrial oxidative stress [262] and enhancing FFAs oxidation, thereby preserving muscle mass in diabetic conditions [263]. Consequently, muscular USP2a primarily contributes to systemic glucose homeostasis by maintaining skeletal muscle mass, a major determinant of whole-body glucose consumption. In contrast, hepatic USP2b aggravates both MASLD and T2DM while simultaneously activating PPARγ [38]. Despite PPARγ activation in both muscle and the liver, the metabolic outcomes differ substantially, indicating that tissue-specific actions of PPARγ underlie these divergent effects. In the liver, PPARγ enhances uptake of circulating FFAs and stimulates de novo lipogenesis, thereby leading to steatosis [264]. The role of hepatic PPARγ in glucose metabolism remains controversial, as liver-specific PPARγ KO mice have been reported to show either improved or worsened glucose tolerance in genetic obesity models [265,266]. These observations suggest that while USP2b-mediated PPARγ activation contributes to steatosis, hepatic USP2b may worsen glucose metabolism through PPARγ-independent mechanisms, such as activation of the C/EBPα-cortisol axis.

## 6. Conclusions

USP2 exerts tissue- and isoform-specific control over energy metabolism: hepatic USP2b drives gluconeogenesis/lipogenesis, whereas hyperglycemia is generally counteracted by USP2a in muscle, brown adipocytes, and macrophages and USP2b in hypothalamic neurons. USP2 also influences cholesterol handling and supports sperm energetics; in cancer, USP2a stabilizes FASN to facilitate lipid acquisition. Human genetic signals linking USP2 to BMI and cross-tissue mechanistic data highlight USP2 as a therapeutic candidate in T2DM, MASLD, and atherosclerosis. Given the context-dependent effects, successful translation will depend on isoform-aware targeting, tissue-selective delivery, and validation in human-relevant models.

## Figures and Tables

**Table 1:** Regulatory roles of USP2 in energy metabolism in various tissues.

Organ/Tissue	Cell	USP2 Isoform	Functional Study Models	Cellular Function	Responses of In Vivo Model	Putative Substrate of USP2	References
Liver	Hepatocyte	USP2b	Mouse cell, Mouse	Promotion of gluconeogenesis	Promotion of hyperglycemia	C/EBPα	[36]
		USP2b	Mouse cell	Promotion of lipid accumulation and cytokine production	Not studied	C/EBPα	[37]
		USP2b	Mouse cell, Human cell, Mouse	Promotion of lipid accumulation	Promotion of fat liver	PPARγ	[38]
		USP2a	Human cell	Promotion of LDL uptake	Not studied	IDOL	[39]
Skeletal muscle	Myoblast	USP2a	Mouse cell	Protection of mitochondrial respiration	Not studied	PGC1α	[40,41]
	Myotube, Myofiber	Unspecified	Mouse cell, Mouse	Protection of mitochondrial respiration	Inhibition of muscular oxidative stress	(-)	[42]
		USP2a	Mouse cell, Mouse	Protect myofiber formation and insulin signal	Inhibition of muscle atrophy	PPARγ	[43]
White adipose tissue	Macrophage	USP2a	Human cell, Mouse	Inhibition of prodiabetic molecules	Inhibition of adipose tissue inflammation	(-)	[44,45]
Brown adipose tissue	Brown adipocyte	USP2a	Mouse cell, Mouse	Promotion of browning adipocytes	Promotion of thermogenesis	EBF2	[46]
Brain (Hypothalamus)	Neural cell	USP2b	Human cell, Mouse	Protection of mitochondrial respiration	Inhibition of sympathetic activation	(-)	[47]
Testes	Sperm	Unspecified	Mouse cell, Mouse	Protection of ATP supply	Maintenance of male fertility	(-)	[48]
	Macrophage	Unspecified	Mouse cell Mouse	Indirect protection of mitochondrial respiration in sperm	Maintenance of male fertility	(-)	[49]
Vascular wall	Macrophage	USP2a	Mouse cell Mouse	Promotion of ferroptosis	Promotion of plaque formation	Keap1	[50]
Cancer	Cancerous cell	USP2a	Human cell	Promotion of fatty acid synthesis	Promotion of cancer growth	FASN	[51,52,53,54]

USP2-expressing organs/tissues and cells, studied USP2 isoforms, studied models, cellular functions, subsequent systemic responses at an individual level, putative substrates of USP2, and references are listed. (-), unidentified. Observations of changes in *USP2*/*Usp2* expression only are not included in this table.

**Table 2:** Metabolic disorders evoked by genetic and chemical perturbation of USP2 in mouse models.

Organ/Tissue	Cell	USP2 Isoform	Types of Intervention	Disorders	Putative Roles of USP2 in Animal	References
Liver	Hepatocyte	USP2b	KD, OE	T2DM	Promotion of hyperglycemia, insulin/glucose tolerance	[36]
			KD, KO	T2DM, MASLD	Promotion of hepatic steatosis, fibrosis, inflammation, insulin/glucose tolerance	[38]
Skeletal muscle	Myofiber	USP2a	KO, OE	Obesity, T2DM, muscle atrophy	Repression of body weight gain, hyperglycemia, insulin/glucose tolerance, muscle atrophy	[43]
		Unspecified	KO	T2DM	Repression of Hyperglycemia, insulin/glucose tolerance	[42]
White adipose tissue	Macrophage	USP2a	OE	Obesity, T2DM	Repression of body weight gain, Hyperglycemia, insulin tolerance	[45]
Brown adipose tissue	Brown adipocyte	USP2a	CB, KD, OE	Obesity, T2DM	Repression of body weight gain, insulin/glucose tolerance	[46]
Brain (Hypothalamus)	Neural cell	USP2b	CB	T2DM	Repression of hyperglycemia	[47]
Vascular vessel	Macrophage	USP2a	KO	Atherosclerosis	Promotion of atherosclerotic plaque formation	[50]

USP2-expressing organs/tissues and cells, studied USP2 isoforms, types of interventions, metabolic disorders of mouse models, putative roles of USP2 in metabolic disorders, and references are listed. CB, chemical blockade; OE, overexpression; KD, knockdown; KO, knockout.

## Data Availability

No new data were created or analyzed in this study.

## Abbreviations

The following abbreviations are used in this manuscript:

AA	Amino acids
AAV	Adeno-associated virus
ABC	ATP-binding cassette transporter
ACSL4	Acyl-CoA synthetase long chain family member 4
ALT	Alanine aminotransferase
AMPK	AMP-activated protein kinase
aP2	Adipocyte protein 2
ApoE	Apolipoprotein E
ApoO	Apolipoprotein O
ARC	Arcuate nucleus
BAT	Brown adipose tissue
11β-HSD	11β-hydroxysteroid dehydrogenase
BMAL1	Brain and muscle Arnt-like protein 1
BMI	Body mass index
CCL	C-C motif chemokine ligand
C/EBP	CCAAT/enhancer-binding protein
C1QTNF3	C1q and TNF related 3
CRISPR	Clustered regularly interspaced short palindromic repeat
DAMPs	Damage-associated molecular patterns
DIM	3,3′-Diindolylmethane
EBF2	Early B cell factor 2
E4BP4	E4-binding protein 4
FABP4	Fatty acid binding protein 4
FASN	Fatty acid synthase
FFAs	Free fatty acids
GEO	Gene Expression Omnibus
GLUT4	Glucose transporter 4
GM-CSF	Granulocyte-macrophage colony-stimulating factor
G6Pase	Glucose 6-phosphatase
GPX4	Glutathione peroxidase 4
GR	Glucocorticoid receptor
HDL	High-density lipoprotein
HFD	High-fat diet
HMGA2	High mobility group AT-hook 2
HuGE	Human genetic evidence
I3C	Indol-3-carbinol
IDOL	Inducible degrader of low-density lipoprotein receptor
IL	Interleukin
iNOS	Inducible nitric oxide synthase
IRS1	Insulin receptor substrate 1
Keap1	Kelch-like ECH-associated protein 1
KO	Knockout
LDL	Low-density lipoprotein
LDLR	Low-density lipoprotein receptor
LH	Lateral hypothalamus
LOX-1	Lectin-like oxidized LDL receptor-1
MASH	Metabolic dysfunction-associated steatohepatitis
MASLD	Metabolic dysfunction-associated steatotic liver disease
Mdm2	Murine double minute 2
MMe	Metabolically activated adipose tissue macrophage
MPS	Microphysiological system
MSR1	Macrophage scavenger receptor 1
mTORC1	Mammalian target of rapamycin complex 1
MURF-1	Muscle ring finger-1
NAFLD	Nonalcoholic fatty liver disease
NAMs	New approach methodologies
NASH	Nonalcoholic steatohepatitis
NF-κB	Nuclear factor-κB
NRF2	Nuclear factor E2-related factor 2
Oct	Octamer transcription factor
OS	Osteosarcoma
oxLDL	Oxidized low-density lipoprotein
OXPHOS	Oxidative phosphorylation
PAI-1	Plasminogen activator inhibitor-1
PCSK6	Proprotein convertase subtilisin/kexin type 9
PDGF	Platelet-derived growth factor
PD-L1	Programmed death-ligand 1
PEPCK	Phosphoenolpyruvate carboxykinase
PET	Positron emission tomography
PER1	Period1
PGC	Peroxisome proliferator-activated receptor gamma coactivator
PNS	*Panax notoginseng* saponins
PPAR	Peroxisome proliferator-activated receptor
PVN	Paraventricular nucleus
qPCR	Quantitative polymerase chain reaction
ROS	Reactive oxygen species
SASP	Senescence-associated secretory phenotype
shRNA	Short hairpin RNA
siRNA	Short interfering RNA
SKP2	S-phase kinase-associated protein 2
SLD	Steatotic liver disease
SREBP	Sterol regulatory element-binding protein
T2DM	Type 2 diabetes mellitus
TGF	Transforming growth factor
TNF	Tumor necrosis factor
UBP	Ubiquitin-specific processing protease
UCP	Uncoupling protein
USP	Ubiquitin-specific protease
VCP	Valosin-containing protein
VMH	Ventromedial hypothalamus
WAT	White adipose tissue
